# The complete mitogenome of the Mountain chicken frog, *Leptodactylus fallax*

**DOI:** 10.1080/23802359.2021.1907809

**Published:** 2021-04-08

**Authors:** Nina F. D. White, Andrew A. Cunningham, Michael A. Hudson, Pablo Orozco-terWengel

**Affiliations:** a Cardiff University School of Biosciences, Cardiff, UK; b Institute of Zoology, Zoological Society of London, London, UK; c Durrell Wildlife Conservation Trust, Jersey, UK

**Keywords:** *Leptodactylus fallax*, mitogenome, phylogeny

## Abstract

The mountain chicken frog (*Leptodactylus fallax*) is a critically endangered frog native to the Caribbean islands of Dominica and Montserrat. Over the past 25 years their populations have declined by over 85%, largely due to a chytridiomycosis outbreak that nearly wiped out the Montserratian population. Within the context of developing tools that can aid in the conservation of the mountain chicken frog, we assembled its complete mitochondrial genome, contributing the first complete mitogenome of the genus Leptodactylus (Genbank Accession number MW260634). The circular genome is 18,669 bp long and contains 37 genes. A phylogenetic analysis reveals that *L. fallax* forms a clade with *Leptodactylus melanonotus*, highlighting the close relationship of Leptodactylus spp. relative to other species from the superfamily Hyloidea included in the analysis.

The mountain chicken frog (*Leptodactylus fallax Müller, 1926*), endemic to the Lesser Antilles, is one of 83 species in the genus Leptodactylus (Frost 2021). As a giant frog with a large appetite, *L. fallax* plays an important ecological role as a native apex predator in its remaining home range on Montserrat and Dominica (Daltry 2002). *Leptodactylus fallax* is critically endangered, largely due to the recent incursion of the infectious disease chytridiomycosis (Hudson et al. 2016). There are thought to be fewer than 100 wild individuals remaining (author’s unpublished observations), so it is important to document genetic features of the species, such as its mitochondrial genome. Genetic records from wild populations allow benchmarks to be set for conservation breeding programmes of *L. fallax*, which could be vulnerable to the loss of genetic diversity in captivity (Lynch and O’Hely 2001). To date, only one, partial mitogenome from the genus Leptodactylus has been described from *Leptodactylus melanonotus* (Zhang et al. 2013). The addition of the *L. fallax* mitogenome improves the genetic record for the genus and may help in the assembly of other Leptodactylus mitogenomes. Here we describe the complete mitochondrial genome of *L. fallax.*

Genomic DNA was extracted from a blood sample taken at Pelican Ghaut, Montserrat (16.777429, −62.170873) in March 2009. The sample is stored in the archive of the Molecular Ecology Lab, Cardiff University School of Biosciences, under voucher ID L2175. DNA was extracted using a QIAGEN DNeasy Blood & Tissue Kit protocol for nucleated blood. Whole genome paired-end Illumina sequencing was performed by Novogene, Hong Kong. A subsample of the raw Illumina paired-end reads was produced using the Novoplasty filter_reads.py script with *L. melanonotus* as a reference mitogenome (Dierckxsens et al. 2016). These reads were assembled in Geneious Prime v.2020.2.4 using the circular de novo assembly function. The longest contig was 19,649 bp using 210,146 reads achieving a mean coverage of 1595.8 (Std. Dev. = 553.8). This contig was run through a circules.py script (Hahn et al. 2013) to check for repeated kmers at either end of the assembly which indicate a circular sequence. A final, circular assembly of 18,669 bp was produced after clipping overlapping motifs at each end of the assembly.

The mitogenome consisted of 42.8% GC, with individual base percentages at: A − 29.7%, C − 28%, G − 14.7%, and T − 27.5%. Annotation was guided using MITOS web server (Bernt et al. 2013), where a total of 37 genes were annotated. 13 protein coding genes, 2 rRNA genes, and 22 tRNA genes were noted alongside a control region (D-loop). The order and orientation of genes that could be compared with the partial mitogenome of *L. melanonotus* were identical.

A maximum likelihood phylogeny was constructed using the 13 concatenated protein coding genes of the mitogenome of *L. fallax, L. melanonotus*, one other member of the family Leptodactylidae (*Pleurodema thaul*), 8 members of the same superfamily Hyloidea, and one distantly related anuran species from the genus Odorrana (Figure 1). Sequences were aligned and trimmed in Geneious, and PHYML was used to construct a maximum likelihood tree with 100 bootstrap replicates. *Leptodactylus fallax* and *L. melanonotus* formed a clade with bootstrap support of 100. The nearest relation based on the alignment was the other member of the Leptodactylidae family, *P. thaul*, and a member of the Telmatobiidae family, *T. chusmisensis*, a Chilean endemic.

**Figure 1.**
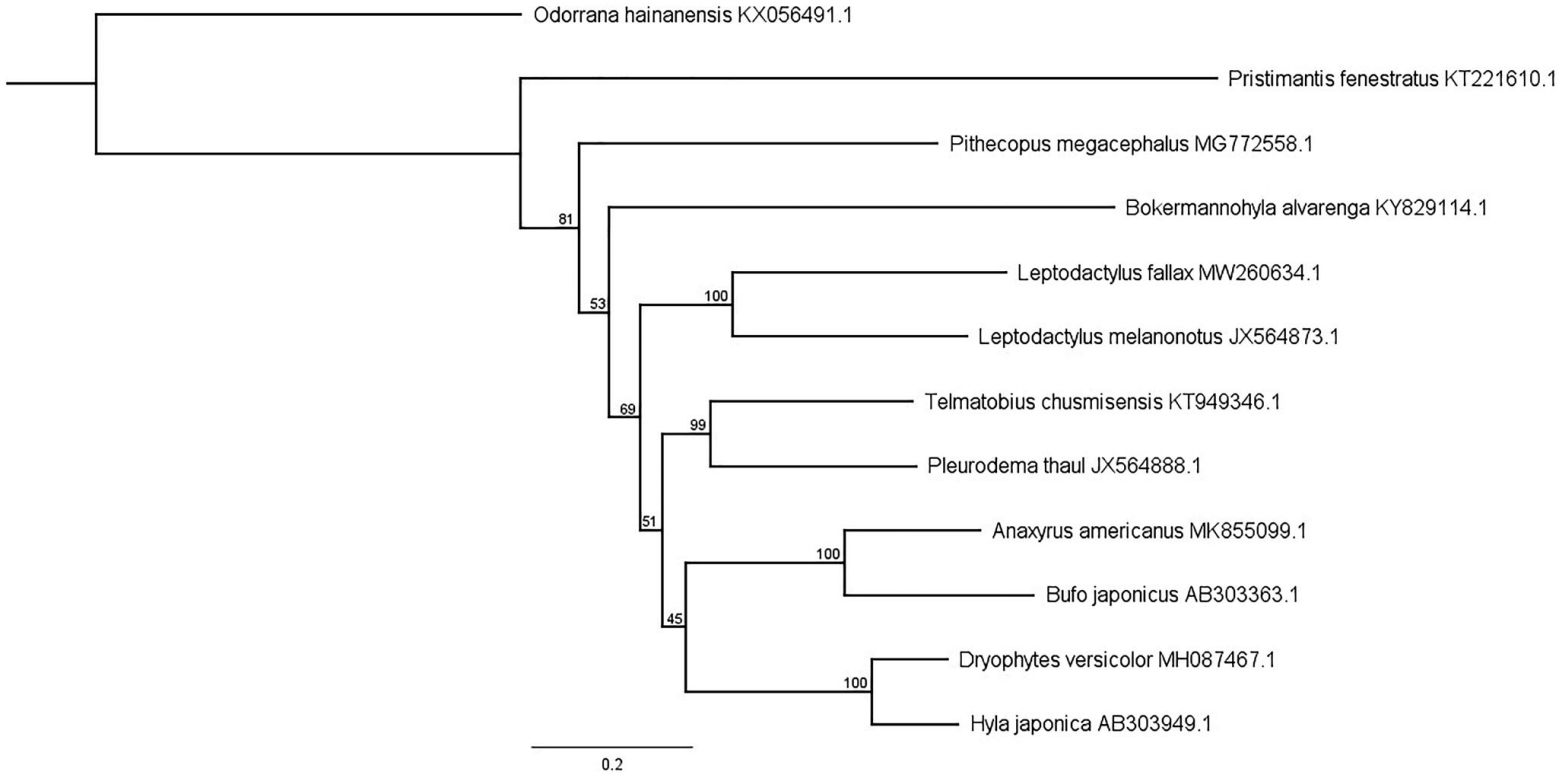
Maximum likelihood phylogeny constructed using a 11,027 bp alignment of mitogenome protein coding genes. Genbank accession numbers are listed after species names. Bootstrap values are listed for each node as a value from 0 to 100. The scale bar represents the number of nucleotide substitutions..

## Data Availability

Mitogenome data supporting this study are openly available in GenBank at https://www.ncbi.nlm.nih.gov/nuccore/MW260634.1 under the accession number MW260634.1. The associated BioProject, SRA, and BioSample numbers are PRJNA705465, SRR13806021, and SAMN18088376, respectively.
